# Neuroplastic changes in addiction

**DOI:** 10.3389/fnmol.2013.00056

**Published:** 2014-01-02

**Authors:** Ildikó Rácz

**Affiliations:** Institute of Molecular Psychiatry, University of BonnBonn, Germany

**Keywords:** addiction, neuroplastic changes, withdrawal, genetic, epigenetics

Drug addiction is a chronic, relapsing disorder, which is caused by many factors including genetic, epigenetic, environmental, and drug-related (Robison and Nestler, 2011). Loss of control over drug intake and compulsion for drug taking are the most characteristic features of addiction (Koob and Volkow, 2010). Although many individuals are exposed to substances of abuse, only subsets enter the addicted state. However, if the addicted state develops, it persists for life, suggesting that the underlying molecular changes in the brain are long-lasting. The present research topic was selected to highlight several new insights about genetic and drug-related factors that contribute to drug-induced neuroplastic changes.

Because dopaminergic cells play a pivotal role in the rewarding action of drugs of abuse, they were the starting point for the study of drug-induced synaptic alterations. Rodriguez Parkitna and Engblom (2012) added new insights to our knowledge about the synaptic plasticity of dopaminergic cells in the ventral tegmental area focusing on NMDA and AMPA receptor functions. For this, knockout mice with selective deletion of the NR1 subunit of the NMDA receptors were used. They examined NMDA receptor plasticity and burst firing activity, and found that this plays an important role in reward learning. Furthermore, they show NMDA receptors on dopaminergic cells are involved in drug-induced associative learning and in recall of drug-associated experiences.

Withdrawal is an unbalanced state characterized by increased stress, anxiety, and depression. During chronic drug consumption and withdrawal, the brain stress response system becomes dysregulated. The expression of several neuropeptides, including corticotropin releasing factor (CRF), neuropeptide Y, and dynorphin are stress-related. Furthermore, these neuropeptides are involved in the modulation of negative emotional states associated with drug addiction (Boutrel and De Lecea, 2008; Allen et al., 2011; Bruijnzeel, 2012).

Yadid et al. in their focused review (Yadid et al., 2012) concentrated on the neuroadaptive processes occurring during withdrawal. Evidence shows that endogenous opioid peptides β-endorphin, enkephalin, and dynorphin play an important role in substance reinforcement. β-endorphin and the neurosteroid dehydroepiandrosterone (DHEA) both modulate mood and drug addiction, and these modulatory functions are linked with each other. Application of exogenous DHEA-S (phosphorylated DHEA) into the nucleus accumbens elevated the level of extracellular β-endorphin. Thus, modulation of DHEA level in the brain may regulate extracellular β-endorphin levels which consequently controls stress coping including mood fluctuations. Together, these processes end up regulating the craving for drugs of abuse.

Dempsey and Grisel (2012) in their research paper examined the role of β-endorphin in the development of locomotor sensitization to repeated chronic alcohol exposure. They found that mice lacking β-endorphin did not develop locomotor sensitization to alcohol. These findings support the notion that β-endorphin modulates the locomotor effect induced by alcohol consumption and contributes to the neuroadaptive changes associated with chronic use.

Haass-Koffler and Bartlett (2012) in their review discussed the role of CRF in alleviation and maintenance of synaptic plasticity in the ventral tegmental area and amygdala. CRF facilitates the molecular changes induced by drugs of abuse like enhancement of glutamate-mediated excitation and reduction of GABA-mediated inhibition. Stress induces plastic changes in the limbic system that are thought to trigger the development of chronic anxiety and loss of control over limited drug use. Regulating stress processes by modulating the function of the CRF system may offer a possible new therapeutic approach in the treatment of relapse.

Besides the stress response, the Dynorphin/κ-opioid receptor (DYN/KOR) system is also highly dysregulated during chronic, excessive alcohol consumption and both contribute to the negative emotional state experienced during withdrawal (Koob and Volkow, 2010). Excessive alcohol consumption leads to the adaptation of the DYN/KOR system at the pharmacological, transcriptional, and epigenetic levels. Various key brain regions are involved via activation of different signaling pathways, like CREB/ΔFosB/BDNF, which contribute to altered downstream events. These changes can lead to escalated alcohol use, anxiety like behaviors, and sensitization following abstinence, which are the most common consequences of alcohol dependence (Sirohi et al., 2012).

Feduccia et al. (2012) in their review added new insights to the function of nicotinic acetylcholine receptors (nAChR) in alcohol and nicotine addiction. The nAChRs are ligand-gated ion channels which are wildly distributed in the brain and play a crucial role in synaptic neurotransmission (Mao et al., 2011). Both alcohol and nicotine are able to activate neuronal nAChRs. Activation of nAChRs by nicotine and alcohol facilitates and maintains long-term potentiation, long-term depression, and also structural changes in the hippocampus, amygdala, and mesolimbic dopaminergic system. Chronic nicotine treatment leads to up-regulation of nAChRs, which serves as a compensatory response to excessive receptor stimulation and is a main contributor to the development of nicotine dependence. Several studies showed that chronic alcohol treatment induces the same processes and also that activation of nAChR function can reduce voluntary alcohol consumption.

Recently, a growing body of evidence shows that epigenetic mechanisms play a pivotal role in long-lasting changes in gene expression by regulation of transcriptional potential (McClung and Nestler, 2003; Chao and Nestler, 2004). Madsen et al. (2012) summarized our knowledge about the epigenetic modulation of gene expression induced by drugs of abuse. Self-administration of drugs of abuse induces transcriptional changes in the cell that represent a key mechanism affecting reward-related learning and further drug-related behaviors. Thus, voluntary drug intake controlled by a fine equilibrium of opposing molecular regulators can facilitate or inhibit compulsive drug use. This research has opened up new therapeutic strategies by modulation of transcriptional regulatory functions.

In her focused review, Kovacs (2012) summarized the role of neuroinflammatory processes in the development of chronic drug-induced molecular changes in the brain. Activation of microglia cells plays a pivotal role in drug-induced morphological, molecular, and physiological changes. These alterations involve release of proinflammatory cytokines, remodeling of synaptic functions, excitotoxic neurochemical changes, and phagocytic activity. Targeting microglia can serve as a potential new treatment strategy in addiction treatment.

Nylander and Roman (2012) provided a summary about the consequences of early-life stress. How changes early in life influence the function of peptide networks, like endogenous opioid peptides, oxytocin, and vasopressin later in adulthood. The results summarized in this review indicate that there is a strong association between early-life rearing conditions, opioids, and ethanol consumption. The effects of ethanol and also the treatment efficacy of opioid antagonists later in life are both dependent on early-life experiences.

Taken together, this research topic delivered new visions to our knowledge about the neuroplastic changes in chronic escalated drug consumption and in the following withdrawal.
